# Pharmacological Therapy of Gastroesophageal Reflux in Preterm Infants

**DOI:** 10.1155/2013/714564

**Published:** 2013-06-26

**Authors:** Luigi Corvaglia, Caterina Monari, Silvia Martini, Arianna Aceti, Giacomo Faldella

**Affiliations:** ^1^Neonatology and Neonatal Intensive Care Unit, S. Orsola-Malpighi Hospital, via Massarenti 11, 40138 Bologna, Italy; ^2^Department of Medical and Surgical Sciences (DIMEC), University of Bologna, 40126 Bologna, Italy

## Abstract

Although gastroesophageal reflux (GER) is a very common phenomenon among preterm infants, its therapeutic management is still an issue of debate among neonatologists. A step-wise approach should be advisable, firstly promoting nonpharmacological interventions and limiting drugs to selected infants unresponsive to the conservative measures or who are suffering from severe GER with clinical complications. Despite of this, a concerning pharmacological overtreatment has been increasingly reported. Most of the antireflux drugs, however, have not been specifically assessed in preterm infants; moreover, serious adverse effects have been noticed in association to their administration. This review mainly aims to draw the state of the art regarding the pharmacological management of GER in preterm infants, analyzing the best piecies of evidence currently available on the most prescribed anti-reflux drugs. Although further trials are required, sodium alginate-based formulations might be considered promising; however, data regarding their safety are still limited. Few piecies of evidence on the efficacy of histamine-2 receptor blockers and proton pump inhibitors in preterm infants with GER are currently available. Nevertheless, a significantly increased risk of necrotizing enterocolitis and infections has been largely reported in association with their use, thereby leading to an unfavorable risk-benefit ratio. The efficacy of metoclopramide in GER's improvement still needs to be clarified. Other prokinetic agents, such as domperidone and erythromycin, have been reported to be ineffective, whereas cisapride has been withdrawn due to its remarkable cardiac adverse effects.

## 1. Introduction

Gastroesophageal reflux (GER) is very frequent in preterm infants. The incidence in those babies born before 34 weeks of gestation approximately amounts to 22% [1]. In the preterm population GER should not be usually considered a pathological phenomenon, as it might be promoted by a number of physiological factors. Among these, are included the supine posture, which enhances the migration of liquid gastric content through the looser gastroesophageal junction, the immature esophageal motility, which leads to a poor clearance of refluxate, and, eventually, the relatively abundant milk intakes [2]. 

The linkage between GER, apneas [3] and chronic lung disease is still controversial [4, 5]. In few cases, however, GER may be associated to clinical complications as, for instance, feeding problems, failure to thrive, esophagitis, and lung aspiration [6], thereby lengthening the hospital stay [7].

The therapeutic management of GER is still debated. A step-wise approach, which firstly promotes nonpharmacological interventions such as body positioning, modification of feeding modalities, or milk thickening, is currently considered an advisable strategy to manage GER in preterm infants [3, 6], limiting drug administration to those infants who do not benefit from conservative measures or with clinical complications of GER [8].

In the last decades, a widespread use of empirical antireflux medications in preterm infants, both during hospital recovery and after discharge, has been reported [9]. Most of these drugs, however, have not been specifically studied in these patients; moreover, antireflux medications have been noticed to cause serious adverse effects. For instance, inhibitors of acid gastric secretion as histamine-2 receptor blockers and proton pump inhibitors (PPIs) have been recently associated with an increased incidence of necrotizing enterocolitis (NEC) [10, 11] and infections [12], whereas a linkage between cisapride administration and QTc prolongation was previously established [13, 14]. Therefore, a careful balance between risk and benefits for each drug should be carried out before starting a pharmacological therapy.

We aimed to provide a complete overview on the pharmacological management of GER in preterm infants, analyzing the evidences currently available conceiving the most prescribed antireflux drugs: surface protective agents as alginate-based formulations, histamine-2 receptor blockers, proton pump inhibitors, and prokinetics.

## 2. Gastroesophageal Reflux: Pathogenesis

Gastroesophageal reflux is very common in early childhood, being particularly frequent among preterm infants [3]. Indeed, several promoting factors may contribute to trigger GER in this specific population [15]. Preterm infants characteristically show a short and narrow esophagus, subsequently resulting in a slight displacement of lower esophageal sphincter (LES) above the diaphragm [16]. As Henry previously disclosed [17], gastrointestinal motor innervation gradually develops as postmenstrual age (PMA) increases. Hence, a nonperistaltic esophageal motility is frequently observed in preterm infants, therefore resulting in a subsequent ineffective clearance of the refluxate from the esophageal lumen [18]. Additionally, esophageal and upper esophageal sphincter (UES) motor responses to an abrupt intraluminal stimulation (i.e., due to the refluxate of gastric content) have been shown to be incomplete before 33-week PMA [19].

Neonates are usually lying in the supine position, which may additionally lead to GER worsening as well as the relatively abundant milk intakes that elicit LES relaxation through the enhancement of gastric distension [2]. 

It has been previously demonstrated that the occurrence of transient LES relaxations (TLESRs) represents the main GER's pathogenic mechanism in preterm infants, being linked to the 92–94% of the overall GER episodes detected in this population [2]. Unexpectedly, no difference was observed in the frequency of TLESRs between healthy infants and those affected by gastroesophageal reflux disease (GERD); however, the latter were disclosed to have a significantly higher proportion of TLESRs associated with acid GER [2]. 

## 3. Gastroesophageal Reflux: Clinical Presentation

In early childhood, the occurrence of GER may vary within a wide range of clinical manifestations, being vomiting and regurgitations the most frequent nonpathological symptoms. Generally, healthy babies who are experiencing frequent regurgitations in the absence of clinical complications are commonly referred as “happy spitters” [20]. 

Other common but less specific symptoms are represented by irritability, sleep disturbances, feeding refusal, or unexplained crying [8], especially if associated with back arching [21]. In fewer, severe cases, GERD may be combined with the presence of spastic torticollis and dystonic body movements, outlining the so-called Sandifer syndrome [22]. 

Sometimes frequent regurgitations or vomiting may be complicated by failure to thrive, despite an adequate caloric intake; thus, diagnosis other than GER, as, for instance, cow's milk protein allergy (CMPA), should be carefully ruled out [3, 8, 23]. 

Furthermore, due to the higher risk of gastric content's aspiration, the occurrence of GER in the neonatal population may contribute to the development of wheezing or pneumonia [24], whereas its linkage with the chronic lung disease is still controversial [4, 5, 25]. 

With regard to the preterm population, the linkage existing between GER, apneas, and cardiorespiratory events represents an actual issue of debate. On one hand, Di Fiore et al. recently reported that the rate of cardiorespiratory events (CEs), defined as episodes of apnea, bradycardia, and desaturations, following GER in healthy preterm infants, is irrelevant when compared to the overall number of events recorded during a 12-hour plethysmographic and pH-MII monitoring [26]. Similarly, no temporal relationship has been previously observed neither between the occurrence of cardiorespiratory events and acid refluxes detected by a pH-probe [27], nor between apneas and GERs recorded by multiple intraluminal impedance (MII) monitoring [28]. 

Conversely, we have previously perceived an increased rate of apneas occurring within the 30 seconds following a GER episode [29]. Moreover, as we have subsequently shown [30], the number of apneas is significantly higher after nonacid GER episodes, which prevail in the early postprandial period [31], confirming Wenzl's previous findings [32]. In accordance with these results, neither thickened formulas [33] nor the administration of sodium alginate [34] was found to improve the rate of apneas in symptomatic preterm infants. Eventually, a significant temporal association between cardiorespiratory events and GER, particularly remarkable among obstructive apneas and MII-GER, has been recently reported by Nunez et al. [35] in a small cohort of both term and preterm infants. Even so, the analysis of piecies of current evidence conceiving the relationship between apneas and GER in preterm infants is partially affected by the small sample sizes and the relevant methodological differences, thereby leaving this issue unsolved. 

## 4. Diagnostic Procedures

The presence of GER, generally suspected on the basis of suggestive clinical symptoms, might be confirmed and characterized by specific diagnostic investigations. Esophageal pH-metry is generally accepted as a standard technique for diagnosing GERD [6], enabling the detection of acid GER episodes, defined by the decrease of intraesophageal pH values below 4, and other parameters as, for instance, reflux index and symptom index. However, a relevant limitation of this technique is its capability of detecting only acid refluxes. Thereby, as the acidity of gastric juice is age-dependent [36] and milk feeds are reported to buffer gastric content's pH [31, 37], pH-metry might result to be flawed in the preterm population.

Multiple intraluminal impedance (MII) monitoring analyses the variations of esophageal electrical impedance through multiple intraluminal electrodes [38]. Due to its specific ability to detect nonacid reflux events, MII monitoring is considered a sensitive diagnostic tool, particularly useful during the postprandial period or in other conditions in which the gastric content is mainly nonacidic [39].

A combined MII and pH monitoring allows to assess acid, weakly acid and alkaline reflux, proximal extent, and nature of the reflux episodes being gas, liquid, or mixed [31, 40, 41], thereby achieving a relevant diagnostic ability. Combined pH-MII has been recognized by the North American Society for Pediatric Gastroenterology, Hepatology, and Nutrition (NASPGHAN) and the European Society for Pediatric Gastroenterology, Hepatology, and Nutrition (ESPGHAN) to be superior to pH monitoring alone for the evaluation of the temporal relation between symptoms and gastroesophageal reflux, particularly if nonacidic, and for the assessment of pharmacological antireflux therapy's effectiveness [23, 41]. Hence, combined pH-MII monitoring is progressively emerging as the best diagnostic choice for GER's detection in preterm infants. 

Nonetheless, even if these diagnostic techniques are highly accurate in detecting reflux events, on the other hand the presence of a probe through LES could potentially contribute to trigger GER episodes [42]. Therefore, therapeutic decisions should be guided by the presence of clinical manifestations and not just on the basis of instrumental GER detection. 

A reflux questionnaire aimed to guide pediatricians' decisions regarding GER's diagnosis and treatment was developed in 1993 by Orenstein et al. [43]. The need of simpler and less invasive tests for diagnosing GERD in the preterm population has recently led Birch and Newell [6] to design a similar reflux scoring system based on clinical observation, adapting the Orenstein's questionnaire for hospitalized preterm infants. As the authors noticed, however, this questionnaire could not supplant the need for standard diagnostic investigations; moreover, it needs to be largely validated before being recommended as a diagnostic tool. 

## 5. Conservative Management

A step-wise therapeutic approach is advisable in the management of GER in preterm infants. Conservative management of GER should be considered the first-line treatment in symptomatic babies who are experiencing frequent vomiting and effortless regurgitations without significant clinical complications. 

On the basis of current evidences, body positioning can be considered a well-established and safe treatment in preterm babies symptomatic for noncomplicated GER, both acid and nonacid [6]. A reduction of GER has been observed in left lateral and prone positions [44–46], whereas right lateral and supine positions were reported to worsen GER [47, 48]. However, due to the risk of sudden infant death syndrome (SIDS) associated to prone position [49], this measure should be restricted to hospitalized infants. 

Furthermore, supplemental benefits can be attained by dietary changes as, for instance, the reduction of feeding flow rate [50] or the use of an extensively hydrolyzed formula [51]. Feed thickening has been found to be almost ineffective in the preterm population [52, 53]. Besides, the concern of a possible association between milk thickening and the development of necrotizing enterocolitis has been raised [54, 55]. Eventually, it should be noticed that a worsening in acid GER's features has been reported after HM fortification [56], while evidencess regarding the effect of nonnutritive sucking [57] and intragastric tubes [42, 49] are still limited and controversial.

## 6. Pharmacological Therapy

The provision of drugs in preterm infants with GER should be taken into account when conservative measures do not provide effective results on GER symptoms, or it might be considered at first instance in those symptomatic infants who are suffering from severe GER clinical complications, as failure to thrive, weight loss despite an adequate caloric intake, hematemesis, aspiration pneumonia, and Sandifer syndrome. We provide a comprehensive analysis of the currently available evidences, regarding the main antireflux medications administered in the neonatal population, with particular reference to preterm infants. 

### 6.1. Alginate-Based Formulations

Alginate-based formulations, acting as a physical protection of the gastric mucosa, are commonly employed to treat GERD, both in adult and pediatric populations. In the presence of gastric acid, sodium alginate precipitates to form a low-density but viscous gel, while sodium bicarbonate, usually contained in these formulations, is converted to carbon dioxide. The latter is entrapped within the gel, forming a foam which floats on the surface of gastric content, preferentially moving into the esophagus instead of acidic gastric contents during GER episodes [58].

With regard to the pediatric population, the first placebo-controlled study, disclosing the effect of sodium alginate on vomiting and regurgitation in symptomatic infants and children, dates back to 1987 [59]. This finding has been subsequently confirmed in an open-label trial [60] testing a sodium alginate liquid formulation at daily doses of 1-2 mL/Kg. Moreover, these comforting data have been eventually proved by Miller [61], who studied a new aluminum-free formula of sodium alginate in infants with recurrent GER, compared to a placebo group. 

On the contrary, Del Buono et al. [62] did not notice any difference in acid GER indexes between an alginate formula and placebo, except for the lower esophageal peaks reached by the refluxate. This opposite result might be explained by the use of a powder formulation, which did not contain bicarbonate, thus mainly exerting a thickening action rather than a buffering one.

Alginate-based formulations are reported to be the most commonly prescribed antireflux medications in preterm infants symptomatic for GER [1]. Despite of this, the evidences currently available on the efficacy and safety of sodium alginate in this specific population are still limited. 

In a previous study [63] we have evaluated the effectiveness of a formulation containing sodium alginate and sodium bicarbonate (Gaviscon Reckitt Benckiser Healthcare), administered 4 times a day at a dosage of 0.25 mL/kg, to improve many GER's features in preterm infants. Sodium alginate decreased the number of acid GER episodes and total acid esophageal exposure, detected by pH-monitoring. Moreover, it also reduced the number of refluxes reaching proximal esophagus, whereas it had no influence on nonacid refluxes, detected by MII. 

The two substances contained in the formulation seem to work together as thickening and buffering factors, exerting a complementary effect in lowering acid GER's indexes. The efficacy of sodium alginate is particularly relevant in decreasing acid GER, which is known to be the most important determinant of GERD [2]. Additionally, due to the bicarbonate buffering effect, GER's pH may probably rise up.

Depending on its physical and chemical characteristics, GER may be classified into acid and nonacid. While the latter occurs in the early postprandial period, when the gastric fullness promotes the passage of gastric content into the proximal esophagus, the former occurs in the late postprandial period, when the stomach is partially empty, and it is suggested to represent the main trigger for reflux-related apneas [64]. The remarkable improvement in acid refluxes suggests that this preparation remains inside the stomach for quite a long period after feeding, also because of the longer time of gastric emptying of preterm infants.

As for safety, drugs containing sodium alginate have been linked to bezoar formation [65] and to adverse events due aluminum's toxicity [66, 67]. Furthermore, the content of sodium within this medication is quite high for preterm infants, thereby potentially leading to hypernatremia. 

The results of our study are in agreement with those disclosed by Atasay et al. [68], who have evaluated the efficacy of a formulation containing sodium alginate and potassium bicarbonate, administered 4 times a day at a dose of 1 mL/kg in a cohort of 41 preterm infants with GERD. Eighty-three percent of the patients with pathologic GER responded to the therapy, showing a significant reduction of acid GER parameters and improving clinical features such as vomiting and weight gain. Moreover, the occurrence of possible side effects as abdominal distension, constipation, diarrhea, thickening of the stool, and anal fissure was also analyzed; none of these manifestations was noticed, except for stool thickening in three infants.

These encouraging but merely preliminary data should be deeply investigated in larger trials, in order to have a complete and faithful scenario particularly regarding the safety profile of sodium alginate in preterm infants. 

As van den Anker [69] suggests in a recent comment on the study by Atasay et al. [68], this background is urgently needed before recommending the routine use of alginate-based formulations in this specific population. 

### 6.2. Histamine-2 Receptor Blockers

Histamine-2 (H_2_) blockers are a group of drugs which compete with histamine for the selective linkage to the H_2_ receptor, placed in the gastric wall. This bond leads to a lowered secretion of the hydrochloric acid by the parietal cells in the stomach and, thus, to an increased intragastric pH [70]. 

Several reports support the effectiveness of H_2_-blockers in children and infants affected by GERD and esophagitis [71–73]. 

Ranitidine is the main H_2_-blocker used in Neonatal Intensive Care Units (NICUs). Like many other medications, it has not been approved by the Food and Drug Administration (FDA) for the use in the preterm population, being therefore prescribed in an off-label manner because of the perceived safety and potential benefits [9]. Ranitidine is frequently administered in a wide range of situations. It is usually employed either as prophylaxis and therapy in preterm infants with stress-induced gastric bleeding [74] or, mostly, in infants with GERD, despite the lack of high-level evidences supporting its efficacy. Ranitidine may be also administered in association with steroids, in order to minimize the risk of gastritis [70]. Nevertheless, the efficacy of H_2_-blockers in the preterm population is still an issue of debate [9].

A research performed in critically ill term and preterm infants, aiming to establish the required optimal dose for these two different populations, proved that ranitidine at the dose of 0.5 mg/kg/twice daily effectively keeps gastric pH over 4 in preterm infants, whereas the optimal dose for term infants amounts to 1.5 mg/kg, three times a day [74]. After the first month of life, oral doses range between 2 and 5 mg/kg twice daily, whereas the intravenous dosage is reported to be 2–4 mg/kg/day, divided in 2 daily doses [75]. However, the chronic use of ranitidine is discouraged, due to the frequent development of tachyphylaxis within 6 weeks from the beginning of the therapy, which leads to a decline of its efficacy [8, 75]. 

With regard to the safety profile of H_2_-blockers, numerous trials have investigated their short run effects on preterm infants [10–12], disclosing no encouraging results. 

As a matter of fact, gastric juice, which is mainly composed by HCl and pepsin, is one of the most important nonimmune protection systems [76], which directly reduces intragastric bacterial proliferation and indirectly modulates the composition of the intestinal microflora [77]. HCl has a powerful bactericidal effect on the exogenous bacteria introduced into the stomach: at pH < 3, gastric juice is able to kill bacteria within 15 minutes [78]. According to this finding, a higher growth of pathogens in the gastroenteric tract has been associated to intragastric pH levels >4 in a cohort of preterm infants [79]. With regard to the effects of H_2_-blockers on gut's bacterial colonization, a lowered fecal microbial diversity and a shift toward a Proteobacteria pattern have recently been disclosed by Gupta et al. [80], therefore potentially predisposing to NEC development. 

The association of gastric acidity inhibitors, such as H_2_-blockers, with a higher incidence of necrotizing enterocolitis and infections in very-low-birth-weight (VLBW) preterm infants represents the most daunting ensue in the current literature. 

Guillet et al. [10] performed a retrospective case-control study on VLBW infants to investigate the association between the incidence of NEC and the use of H_2_-blockers, as ranitidine, famotidine, and cimetidine. A significant linkage has been proven, with an overall incidence of NEC of 7.1%. In particular, the administration of these drugs started at a mean of 18.9 ± 15.5 days before NEC development. These data have been recently confirmed by Terrin et al. [12], who have acquired information about VLBW infants from four different Italian NICUs. The patients were clustered into two different groups: infants treated with ranitidine as prophylaxis or treatment for stress-induced peptic disease or suspected GERD, and infants not exposed to this drug, as control cohort. According to their results, NEC was more frequent in infants treated with ranitidine (rate 9.8%) compared to those who did not receive it (rate 1.6%), although the risk of NEC was not associated neither with the dose nor with the duration of treatment. Moreover, the authors documented a higher rate of infections (overall infections, sepsis, pneumonia, and urinary tract infections) and fatal outcome in the treated VLBW infants. 

The latest evidence on the linkage between H_2_-blockers and NEC has been provided by Bilali et al. [81] in a case-control trial: the authors documented a higher incidence of NEC in preterm infants treated with ranitidine when compared to the control group (17.2% versus 4.3%, resp.).

Moreover, the provision of H_2_-blockers has been reported to strike down several leukocyte's functions, thus leading to an insufficient control of the production of inflammatory cytokines in the intestinal tract [82, 83]. Therefore, the factors mentioned above contribute importantly to increase the risks of infections. According to these findings, Canani et al. [84] demonstrated a more frequent onset of infections in children aged 4–36 month, symptomatic for GERD, and treated with GA inhibitors. In particular, a significant higher rate of acute gastroenteritis and community-acquired pneumonia was observed. These findings were probably due to hypochlorhydria, induced by an 8-week treatment with ranitidine, at a daily dose of 10 mg/kg, or omeprazole, at a dosage of 1 mg/kg/day. 

Stoll et al. [85] demonstrated an increased rate of bacteremia, late onset sepsis, and meningitis in VLBW treated with both H_2_-blockers and postnatal steroids, in order to prevent the risk of gastrointestinal bleeding. 

A previous analysis of the risk factors for the development of bloodstream infections in a cohort of both term and preterm newborn admitted to NICU registered a highly significant association with H_2_-blockers' administration [86]. 

H_2_-blockers are probably overused in most of the NICUs to treat many clinical conditions, without any evidence of benefits, and mostly burdened by an adverse risk-benefits ratio.

### 6.3. Proton Pump Inhibitors

PPIs act as long-term blockers of the gastric proton pump, which catalyzes the final phase of the acid secretory process, hindering both basal and stimulated acid secretion by the parietal cells. 

Data collected by MII in preterm and term infants with GERD showed that PPIs increase the esophagus baseline levels of impedance, which is known to be related to the esophageal mucosal integrity [87], suggesting an ameliorative effect. 

The prescription of PPIs as therapeutic agents for the treatment of GERD in the pediatric population has largely increased over the last 10 years, in particular after the therapeutic failure of H_2_-blockers [88]. Currently available PPIs, however, are not approved for being prescribed below one year of life, with the exception of esomeprazole, which has recently gained the indication for the short-term treatment of erosive esophagitis in infants from 1 to 12 months of age. 

Data on the safety and efficacy of PPIs in the preterm population are few and controversial. The effectiveness of omeprazole on preterm infants with GERD has been investigated by Omari et al. [89]. This drug, administered at a daily dose of 0.7 mg/kg, yielded a significant decrease of acid GER frequency and of the overall degree of esophageal acid exposure, which fell even below the currently defined normal levels. However, despite this clear pharmacodynamic effect, omeprazole appeared clinically ineffective to relieve GER symptoms, confirming the previous finding of a double-blind placebo-controlled trial, performed on infants aged 3 to 12 months [90]. 

Similarly, Orenstein et al. [91] assessed the efficacy of lansoprazole versus placebo on a large cohort of both term and preterm symptomatic infants, showing no significant advantage over placebo in the reduction of symptoms attributed to GERD (i.e., crying, regurgitation, refuse of feeding, back arching, wheezing, and coughing). Besides, a trend towards increasing serious adverse effects was reported in the lansoprazole group, regarding, in particular, lower respiratory tract infections. However, as the enrolled infants did not undergo a pH-MII evaluation, the authors hypothesized a causal role of predominant nonacid reflux events, for which PPIs are ineffective, on GER symptoms. 

On the contrary, a recent study by Omari et al. [92], on the effectiveness of esomeprazole in preterm infants, demonstrated a significant decrease in the number of GERD-related symptoms, a remarkable reduction of the overall esophageal acid exposure and, as previously found [93], a lowered number of acid bolus reflux episodes whereas, as expected, nonacid GER features were not influenced. However, these results were not controlled for placebo effects; therefore, they should be confirmed in further placebo-controlled trials.

With regard to pantoprazole, a daily dose of 1.2 mg/kg has been recently reported to improve the frequency of acid GER as well as its mean clearance time in both term and preterm infants. Nonetheless, adverse effects were perceived in more than half of the cohort, being anemia, hypoxia, and constipation the most frequently observed [94, 95]. However, as preterm infants were not analyzed separately from term infants, the specific role of pantoprazole on this specific population cannot be currently ascertained.

PPIs are known to decrease gastric mucosal viscosity [96], to reduce gastrointestinal motility, and to delay gastric emptying [97], potentially enhancing the growth of pathogenic bacteria and leading to a disruption of gut microbiota [98]. Moreover, PPIs have been shown to inhibit neutrophils' chemotactic migration [99], to constrain their phagocytic activity [100], and to decrease the adherence of these cells to the endothelium [101], consequently leading to an increased risk of bacterial infections. According to the issues described so far, a higher incidence of intragastric bacterial infections [102] and community-acquired pneumonia has been reported in association with PPIs' therapy [103]. As mentioned above, children with gastric acid suppression, induced both by PPIs and H_2_-blockers, showed a higher incidence of community-acquired pneumonia and gastroenteritis [84]. 

As asserted in a recent systematic review, a higher incidence of NEC has been reported in preterm VLBW infants in association with the suppression of gastric acidity, induced both by H_2_-Blockers and PPIs [11]. The state of gastric hypochlorhydria, induced by acid suppression, may allow bacterial survival, enhancing gut colonization and potentially leading to bacterial overgrowth, which is known to play an important role in the pathogenesis of NEC [80]. Additionally, it should be considered that gastric juice becomes more acid as gestational and postnatal age increases [36]. Therefore the administration of gastric acidity inhibitors in preterm infants, who already have a lower gastric acidity, will make them more susceptible to bacterial overgrowth, potentially enhancing the risk of NEC development. 

So far, it is not possible to fit these evidences specifically for PPIs, as data currently available on the occurrence of NEC and infections are jointly concerning both PPIs and H_2_-blockers. 

Hence, further systematic and controlled assessments should be carried out to clarify the clinical efficacy of PPIs on GERD's symptoms and their safety in the preterm population. On the basis of the present evidences, pharmacological therapy with PPIs seems to result in an adverse benefit-risk balance; therefore, it is not routinely recommended in preterm infants with symptomatic GERD.

### 6.4. Prokinetic Agents

Promotility agents (cisapride, metoclopramide, erythromycin, and domperidone) belong to a family of drugs which have been widely employed in pediatric practices, in order to reduce the symptoms of GER [104].

In particular, these drugs seem to improve gastric emptying, to reduce emesis, and to enhance LES tone, thus allowing to treat clinical features of GER [105]. 

#### 6.4.1. Cisapride

Cisapride is the most largely investigated prokinetic drug, being used as a treatment of GER in adults, children, and neonates. 

Cisapride is able to enhance the release of acetylcholine from the mesenteric plexus [13], therefore decreasing GER. However, this medication seems to be an important antagonist of the rapid component of the delayed rectifier current of potassium in cardiac cells, thus acting as a III class antiarrhythmic medicament [13, 105].

The clinical efficacy of cisapride in reducing GER in preterm infants has been demonstrated by Ariagno et al. [106]. The authors found a significant reduction in reflux indexes and in the number of GER episodes lasting more than 5 minutes, whereas the therapy was ineffective on the total number of refluxes/24 hours and on the duration of the longest episode. 

On the contrary, McClure et al. [107] raised concerns on the efficacy of cisapride in preterm infants, as it was observed to cause a delay in gastric emptying, which led to an amplification of refluxes and their symptoms. Therefore, the authors did not recommend its use in this particular population.

As the metabolism of cisapride occurs through the cytochrome P 450 (CYP 450) system, which is not fully developed in preterm infants, the simultaneous provision of other drugs inhibiting the CYP 450, such as azole antifungals and macrolides, may further reduce cisapride clearance, increasing its serum levels and, therefore, resulting in a major toxicity [13, 106].

Due to its cardiac effects, the relationship existing between the administration of cisapride in preterm infants and the prolongation of QTc interval has been deeply investigated.

A prolongation of QTc interval in infants and children receiving cisapride has been previously reported by several authors [108, 109]. Semama et al. [110] confirmed a significant increase in the QTc interval in a cohort of term infants treated with cisapride at the dose of 0.2 mg/kg 4 times a day; in particular, the prolongation of the interval resulted to be dose dependent, probably due to the immaturity of liver enzymes which leads to an accumulation of cisapride.

With regard to the preterm population, as Dubin et al. have demonstrated, 48% of the infants treated with cisapride developed anomalies of repolarization; QTc values were significantly longer, especially in babies with gestational age lower than 32 weeks [13].

In a previous study [14], we have examined the possible existence of a relationship between fetal growth and QT prolongation, in a cohort of preterm infants receiving cisapride compared to a control group. In relation to the fetal growth pattern, the infants enrolled were classified as adequate-for-gestational-age (AGA) or small-for-gestational-age (SGA). Both baseline QTc and in-treatment QTc were significantly higher in the SGA group when compared to the values of AGA infants. Therefore, according to these results, intrauterine growth retardation might represent a risk factor for cisapride-inducted QT interval's prolongation in preterm infants.

Hence, due to the possible cardiac toxicity of cisapride and the increased risk of potentially lethal cardiac arrhythmias or sudden death, cisapride has been gradually withdrawn [111], and it is no longer an approved therapy for GER. 

However, if an isoform of this medicament, which has no cardiac side effects, becomes available, more detailed studies should be initiated, in order to investigate the real effects of cisapride on GER and its clinical features. 

#### 6.4.2. Domperidone

Domperidone is a peripheral dopamine D_2_-receptor antagonist, commonly provided to treat regurgitation and vomiting. As a matter of fact, it is able to enhance motility and gastric emptying and to reduce postprandial reflux time [112].

To date, there are few evidences of its efficacy in infants and children with GERD [113, 114], and none in preterm infants [6]. In their review dated 2005, Pritchard et al. [112] demonstrated no convincing efficacy of domperidone in the treatment of GER or GERD in young children, mainly because of several limitations, such as the small number of trials or the high methodological heterogeneity in the studies analyzed. In fact, domperidone does not seem to be more effective in improving symptoms of GER compared to placebo [113]. Recently, Scott [114] confirmed the above mentioned findings, showing little convincing evidence for the efficacy of domperidone in infants with GER. A recent study by Cresi et al. [115] aimed to assess the effectiveness of domperidone on both term and preterm infants symptomatic for GER. The authors showed a paradoxical increase in the number of GER episodes as well as a reduction of their duration, whereas no effects were found in height and pH of refluxes. As hypothesized by the authors, domperidone may amplify the motor incoordination of neonatal gastroesophageal tract. Therefore, the efficacy of this drug in the management of neonatal GER still appears controversial.

Despite no side effects have been reported in all the four trials, domperidone might provoke serious neurologic symptoms, such as extrapyramidal symptoms, oculogyric crises, and long-term hyperprolactinemia [112]. The pediatric population is particularly susceptible to these problems, due to an immaturity of the nervous system and blood-brain barrier.

Moreover, domperidone, such as cisapride, is metabolized by the cytochrome P450; the immaturity of this system, or the simultaneous provision of drugs, which may inhibit its functionality, may lead to higher concentrations of this medicament, consequently enhancing its toxicity.

#### 6.4.3. Erythromycin

Erythromycin, a common used macrolide antibiotic, acts as a strong nonpeptide motilin receptor agonist that contributes to enhance gastric emptying and induces phase III activity of the interdigestive migratory motor complex (MMC), propagating from the stomach to the ileum [116]. Erythromycin increases the release of endogenous motilin and stimulates cholinergic nerves of the gastrointestinal tract, thus resulting in a major release of calcium and in the contraction of muscles of the gut [117].

Oral erythromycin has been proposed as a rescue medicament for feeding intolerance [118]. Specifically, three different oral doses have been investigated: a high dose (12.5 mg/kg administered 4 times a days for an overall period of 14 days) [116], an intermediate dose (10 mg/kg administered 4 times a day for 2 days followed by 4 mg/kg 4 times a day for the next 5 days) [119], and finally a low dose (6–15 mg/kg/die) [118, 120]. Although an improvement of gastrointestinal dysmotility, as well as a reduction of days gained to establish an adequate enteral nutrition, has been reported in these trials, the action of erythromycin in promoting enteral feeding appears to be dose as well as age-dependent. In fact, a decrease of the effectiveness of this medication has been observed in the more preterm infants (<32 weeks of gestational age), probably due to gut immaturity [117]. 

Recently, a large randomized controlled trial demonstrated in a preterm cohort a significant improvement on parenteral-nutrition associated cholestasis [121]. This finding may be justified by the quicker attainment of full enteral feeding, at the intermediate-dose of erythromycin (5 mg/kg 4 times/day for 14 days), therefore resulting in a shorter duration of parental nutrition [121, 122]. Regarding the erythromycin's effectiveness on GER, one of the mentioned trials [116], performed in a small number of preterm infants, reported no significant improvement in GER indexes after the low-dose provision. 

Possible adverse effects have been observed in relation to erythromycin's administration. Among them, an increased risk of infantile hypertrophic pyloric stenosis has been reported, especially in association to an early use, that is, during the first 2 weeks of life [123]. Moreover, cardiac arrhythmias have been related to erythromycin's intravenous administration [124]. 

#### 6.4.4. Metoclopramide

Metoclopramide is a dopamine agonist, which improves the responses of the upper gastrointestinal tract to acetylcholine [125]. Moreover, metoclopramide has been previously shown to enhance LES tone [126].

Therefore, thanks to its promotility properties, metoclopramide has been widely used as treatment of GERD in infants and children, despite the lack of rigorous evidences approving its usage [127].

Because of its widespread employment and an increasing number of concerns about its toxicity in infants, Hibbs and Lorch [127] carried out a systematic review regarding the provision of metoclopramide for GERD in infants aged 0 to 23 months. Twelve studies, testing metoclopramide at doses ranged between 0.1 and 1 mg/kg, were evaluated. Conversely to a Cochrane review published in 2004 [128], which affirmed the effectiveness of metoclopramide in reducing both clinical symptoms and reflux indexes in infants with GERD, the conflicting results of the studies and the lack of a valid demonstration of the metoclopramide‘s efficacy or toxicity did not allow the authors to assess a risk-benefit profile of metoclopramide in infants affected by GERD. However, only few studies evaluated in this review had been performed in the preterm population. 

Another trial performed in preterm infants regarding metoclopramide's effectiveness failed to demonstrate the improvement of bradycardia clinically attributed to GER [129].

Eventually, metoclopramide's administration might be associated to adverse effects [130]; particularly, irritability was the most frequent side effect, followed by dystonic reactions, drowsiness, oculogyric crisis, emesis, and, eventually, apnea. 

Therefore, the current literature is insufficient to either support or contrast the employment of metoclopramide in the usual GERD's treatment.

## 7. Conclusions

Although GER is a very common condition among preterm infants, its therapeutic management in this peculiar population still remains controversial.

A step-wise therapeutic approach, primarily based on nonpharmacological strategies, should be advisable in the management of preterm infants affected by noncomplicated clinical GER, especially in the so-called “happy spitters” [8]. When conservative measures do not provide effective results, or in the presence of clinical complications, the provision of a pharmacological therapy should be considered. 

Although the empirical prescription of antireflux drugs in preterm infants affected by GERD is widespread [9], the overall available evidences regarding the efficacy and the safety of antireflux drugs in the preterm population are quite limited. As a matter of fact, most of these medications have not been neither assessed nor approved for being used in preterm infants. Additionally, serious side effects have been reported in association to their provision. 

On the basis of preliminary results, alginate-based formulations might be considered a promising treatment of GERD, both buffering the gastric content and physically hampering the refluxate. However, further trials are advisable in order to confirm these findings and, in particular, to test out the safety of these medications before recommending their routine use. With regard to inhibitors of gastric acidity, as H_2_-blockers or PPIs, evidences conceiving their effectiveness in preterm infants with GERD are limited. Furthermore, a significantly increased risk of NEC and infections has been noticed, therefore leading to an unfavorable risk-benefit ratio. Due to conflicting evidences, the efficacy of metoclopramide in GERD's improvement is still controversial. Other prokinetic agents, such as domperidone and erythromycin, have been reported to be ineffective, whereas cisapride, largely used to treat GERD in the preterm population up to a decade ago, has been withdrawn due to its remarkable cardiac adverse effects. 

Hence, to avoid a harmful overtreatment in the preterm population, pharmacological therapy should be limited to selected infants suffering from GER complications or after the failure of the conservative management. Finally, the therapeutic choice among the several antireflux medications currently available should represent the result of a careful and targeted risk-benefit balance. 
